# The Restorative Effect of the Natural Environment on University Students' Psychological Health

**DOI:** 10.1155/2020/4210285

**Published:** 2020-05-08

**Authors:** Emma A. Payne, Natasha M. Loi, Einar B. Thorsteinsson

**Affiliations:** University of New England, Armidale, Australia

## Abstract

The present study evaluated the effect of a three-week intervention aimed at improving psychological health in university students. Participants included 200 Australian students randomly assigned to an experimental or waitlist control group, with 42 adhering to intervention instructions. Participants in the experimental group read a story about someone who used the natural environment to decrease stress and burnout levels and to increase their perceived satisfaction with life. They were then instructed to spend 20 minutes each week, for three weeks, in any chosen natural environment. Waitlist control participants received intervention instructions three weeks later. Restorativeness was positively associated with life satisfaction and negatively related to stress and burnout. Experimental participants, compared to waitlist control participants, experienced a significant decrease in stress; however, the intervention had no effect on life satisfaction or burnout. More research is still needed to determine the practical significance of nature exposure on university students' psychological health.

## 1. Introduction

Spending time in nature has long been associated with positive feelings. This notion has been evident for centuries, whereby historical accounts from both Eastern and Western cultures have illustrated traditions linking the outdoors to feelings of peacefulness and tranquillity [1]. Recently, researchers have focused on the impact nature has on our psychological health, particularly the role it plays in reducing elevated levels of stress (e.g., [2–4]). Stress, depression, and anxiety are common problems faced by university students [5], as they may find themselves navigating through competing demands and increased responsibility while at university [6]. This can be both challenging and stressful and may manifest in mental health problems such as burnout and decreased life satisfaction [7]. Furthermore, increased stress may contribute to poor coping approaches and poor sleep quality leading to fatigue [8], thus potentially leading to further feelings of burnout.

Experimental studies have found strong evidence linking natural environment exposure to recovery from stress [3, 4]. Consistent with this research, some researchers have suggested that spending even a small amount of time in a natural setting can result in improvements in psychological health [9, 10]. Locations that allow personal adaptive resources to be renewed when faced with everyday demands are called *restorative environments* [11].

## 2. Psychological Health in University Students

Student distress can be characterized in terms of elevated levels of stress and burnout. Stress is experienced when an individual does not possess the psychological, biological, or social capacity to meet the demands of a given situation. In educational settings, excessive stress over a prolonged period can result in burnout—a state of complete mental, physical, and emotional exhaustion [12]. Burnout among university students may be related to feelings of exhaustion due to study burdens, having a pessimistic and apathetic outlook concerning one's study, and feeling incapable as a student [12]. Burnout research has focused largely on healthcare fields, in particular, medical students [13] where it has been shown to be closely associated with thoughts of discontinuing studies [14] and with suicide [15]. While research suggests that natural environment intervention settings are beneficial for those suffering burnout [2, 16], few experimental studies to date have investigated this effect on a university student sample. This is particularly relevant as university students are faced with increased levels of burnout compared to the general population [7].

Howell et al. [17] conducted a study of 452 introductory psychology students attending an urban university in Canada. The study examined the association between nature connectedness and life satisfaction, which was included as part of a well-being measure. The researchers found significant positive correlations between connectedness with nature and psychological well-being. However, this study did not actually examine whether physically spending time in nature resulted in increased well-being. Research has indicated that students who experience greater life satisfaction are more resilient and emotionally stable in the face of academic challenges [18]. Conversely, students who indicate lower levels of life satisfaction experience diminished focus and poorer academic performance at university [18].

A correlational study of adults with traumatic-onset spinal cord injury (*N* = 650) demonstrated that access to the natural environment was positively related to satisfaction with life at one year postinjury, showing a large effect size, *R*^2^ = 0.28 [19]. Such findings demonstrate that a positive link between exposure to nature and satisfaction with life exists in the literature.

## 3. The Relationship between Restoration and Nature

At its most fundamental, restoration refers to the action of returning something to its former or original condition [20]. In the context of the present study, the aim was to examine how exposure to natural environments may facilitate restoration of an individual's psychological health. There are two prominent theoretical perspectives that offer an explanation and have guided research, as to why natural environments best serve restoration of psychological health: stress recovery theory (SRT; [4, 21]) and attention restoration theory (ART; [22]). The present study focuses on the commonality between these theoretical frameworks which both identify nature as the optimal restorative environment (e.g., [4, 23]).

SRT falls within a psychoevolutionary framework. This perspective contends that because human evolution has predominantly occurred in natural environments, individuals are to some degree physiologically and perhaps psychologically more able to adapt to natural as opposed to urban environments [23]. Conversely, ART is embedded within a psychofunctionalist framework. Here, humans possess an unlearned predisposition to be responsive and react positively to natural content that was favourable to survival during evolution [24]. Although both theories identify nature as the optimal restorative environment, they disagree on the primary factor that drives the individual toward a restorative setting. In SRT, it is *physiological stress,* that is, any external or internal condition that disrupts the homeostasis of a cell or organism [25], and in ART, it is *mental fatigue,* when an individual experiences a period of low attention capacity or cognitive impairment, typically associated with prolonged mental activities or stress [26].

Research led by SRT normally measures physiological stress before and after exposure to different settings. For example, Tsunetsugu and colleagues [10, 27] measured the physiological effects of viewing urban forest landscapes in real life. The findings showed that different environments (forested vs. urban areas) had different impacts on physiological measures. In particular, sympathetic nervous activity was significantly lower and parasympathetic nervous activity was significantly higher when participants were exposed to the forested areas. The researchers also included a measure of affective state, finding that the physiological and psychological outcomes were generally consistent; this supports the notion that physiological and psychological stress reactions are interrelated and do not occur in isolation [28].

Research related to these theories overlaps on two key findings: (1) natural settings are commonly considered to be more restorative than urban or artificial settings; and (2) when individuals are in greater need of restoration, their preference toward different environments will be affected (i.e., they will be more inclined to natural versus urban environments; [11, 29]). The inclination toward natural environments is explained by the fact that restoration occurs more easily in these settings. People experiencing mental fatigue give higher preference to natural as opposed to urban environments [24].

However, studies guided by SRT and ART frameworks are limited as their results have not been compared to a waitlist control condition [2]. It is common for experiments within the environmental psychology field to compare relaxation in a natural environment to that of an urban environment. However, there can be a tendency to regard the categories of “natural” and “urban” as being more clearly defined than may be the case in everyday life. This is because the way in which people experience different kinds of natural settings may differ. For example, in a study highlighting the importance of water in stimuli selection, White et al. [27] point out that many studies in this field have demonstrated a bias toward the inclusion of aquatic scenes in the positive-natural category and that urban scenes containing water were just as likely to elicit positive responses. Such groupings may be appropriate from a land-use perspective but may be far less useful as typologies of natural settings [22, 30, 31]. Thus, there is a need for research comparing individuals exposed to a natural environment to those not exposed to a natural environment and to determine whether this affects psychological health.

## 4. Study Objectives

The current study aimed at examining the relationship between restorativeness, well-being, and student distress. Given the literature to date, it is evident that university students experience stress which can manifest as stress-related illnesses such as burnout and decreased life satisfaction [6, 7] and that nature can be restorative (e.g., [2, 3, 32]). It was therefore hypothesized (Hypothesis 1) that higher levels of restorativeness would positively correlate with well-being (life satisfaction) and negatively with distress (stress and burnout).

The primary objective of this study was to determine whether spending time in nature would increase psychological health in university students. Natural environment interventions have been particularly useful in establishing that exposure to the natural environment improves psychological health outcomes. However, no natural environment interventions have yet, to our knowledge, measured the effect of nature exposure on burnout and satisfaction with life in a university student population. These measures of psychological health are particularly relevant as students can experience substantial levels of burnout during their time at university [7]. Furthermore, decreased satisfaction with life is associated with low academic performance and diminished focus in students [2, 18, 27, 30, 31]. It was therefore also hypothesized (Hypothesis 2) that participants who spent time relaxing in or simply taking in the natural environment would show decreased levels of stress and burnout and increased levels of life satisfaction compared to a waitlist control (after controlling for prescores).

## 5. Method

### 5.1. Participants

The initial questionnaire was completed by 37 male and 163 female participants, ranging in age from 18 to 68 years (*M* = 31.20, SD = 11.84). Inclusion criteria specified males and females over 18 years of age, individuals who were currently enrolled in tertiary studies, and individuals who speak English. Fifty-eight (29%) participants indicated that they were currently enrolled in their first year of undergraduate study, 26 (13%) were in their second year, 39 (19.5%) were in their third year, 39 (19.5%) were enrolled in an Honours program, 20 (10%) were in a Master's program, 3 (1.5%) were currently completing a PhD, and 15 participants (7.5%) selected the “other” category, providing answers such as “Graduate Diploma” and “Combined Masters and PhD.”

There were 126 (63%) participants who indicated that they currently resided in an urban location and 64 (32%) who indicated that they resided in a rural location. Ten (5%) of the participants answered “other” indicating, for example, that they resided in a “remote area”. Most participants (77.5%) indicated that they were of Australian nationality. This was followed by 9% European, 6.5% Asian, 3% North American, and the categories: South American, African, others, and prefer not to say each contained 1% of the participants. The baseline characteristics of participants in the experimental and control groups are presented in Table 1.

## 6. Materials

### 6.1. Demographics

Participants were asked for their sex, age, ethnicity, location (i.e., rural, urban), their fields of study, and the number of years they had been enrolled in tertiary study.

### 6.2. Restorativeness

The Restorative State Scale (RSS; [32]) examines changes in restorative state over time. The scale includes items that capture overall experience (e.g., “I feel connected to the natural world”), as well as items that assess more distinct functions of the restorative nature experience (e.g., “My mind is not invaded by stressful thoughts”). The nine items ask participants to think about a natural setting that they had visited in the last week and to remember how they felt in that environment. Respondents are asked how applicable each item is, with response options ranging from 1 (*do not feel at all*) to 7 (*feel very strongly*). Scale scores are derived by averaging the responses. The RSS has previously demonstrated good reliability (*α* = 0.79; [32]). Internal reliability for the current sample was also good (*α* = 0.78 at preintervention).

### 6.3. Stress

The 10-item Perceived Stress Scale (PSS-10; [33]) measures how frequently stress-related feelings and thoughts occurred during the past month (e.g., “In the last month, how often have you felt that you were effectively coping with important changes that were occurring in your life?”). Responses are scored on a 5-point Likert scale ranging from 0 (*never*) to 4 (*very often*). Negatively worded items are reverse-scored prior to obtaining the total. Scores range from 0 to 40, with higher scores indicating greater perceived levels of stress. The PSS-10 has evidence of good reliability, as well as convergent validity against other measures of anxiety and depression [34]. Internal reliability for the current sample was good (*α* = 0.89 at preintervention).

### 6.4. Burnout

The Maslach Burnout Inventory—Student Survey (MBI-SS; [12]) is a 15-item scale that measures academic burnout. It includes three subscales: (1) emotional exhaustion (e.g., “I feel burned out from my studies”), (2) cynicism (e.g., “I have become less enthusiastic of my studies”), and (3) academic efficacy (e.g., “During class I feel confident that I am effective in getting things done”). High exhaustion and cynicism, combined with low efficacy, indicate burnout. Responses from the MBI-SS are scored on a 7-point scale ranging from 0 (*never*) to 6 (*always*). Negatively worded items are reverse-scored before deriving the overall scale mean, ranging from 0 to 6. Higher scores indicate greater levels of burnout. The MBI-SS showed good reliability (subscale *α*s > 0.80), as well as convergent validity against other student burnout measures [35]. In the current sample, the MBI-SS had a Cronbach's alpha of 0.89 at preintervention.

In the present study, some items on the MBI-SS were adapted to ensure the applicability of the questionnaire to online (off-campus) students as well as on-campus students. For instance, “I feel used up at the end of a day at university” was changed to “I feel used up at the end of a day at, or studying for, university”.

### 6.5. Life Satisfaction

The Satisfaction With Life Scale (SWLS; [36]) measures global life satisfaction (e.g., “I am satisfied with my life”). The five items are scored on a 7-point Likert scale ranging from 1 (*strongly disagree*) to 7 (*strongly agree*). Scores range from 5 to 35, with higher scores signifying greater life satisfaction. A meta-analysis comprising 62 studies demonstrated that the mean SWLS reliability was adequate (*α* = 0.78; [37]) and that the SWLS displays convergent validity against other well-being measures [38]. Internal reliability for the current sample at preintervention was good (*α* = 0.89).

### 6.6. Procedure

Approval to conduct the present study was granted by an Australian university's Human Research Ethics Committee (HE17-064). The survey was built using Qualtrics™ software (Provo, UT) and made available via a link on social media sites, via the university's first-year psychology student pool, and by advertisement through other Australian universities. Participants first read an information sheet which outlined the purpose of the study and were informed that their responses were confidential and anonymous and that withdrawal from the study was permitted at any time. Participants also provided implied consent. They then provided demographic information before completing the RSS, PSS-10, MBI-SS, and SWLS before being randomly assigned via a function in Qualtrics™ which allocated participants to either the experimental or waitlist control condition. Participants in the experimental group read a vignette developed for the current study about a fictional character, Rebecca, who experienced a decrease in feelings of stress and burnout and increased feelings of satisfaction with life, after spending time relaxing in the natural environment. The vignette was designed to provide participants with context to what the intervention involved. In addition, this vignette was provided to help motivate participants as well as highlighting the benefits of engaging in the intervention. Experimental participants were then (1) instructed to spend at least 20 minutes each week, over a period of three weeks, in any chosen green or natural habitat and (2) encouraged to create a physical or electronic reminder to do so. The 20-minute timeframe was chosen as the previous research has found that a significant positive effect on well-being can occur after spending as little as 15 minutes in a natural environment (e.g., [39]). To ensure consistency, participants were asked to complete the intervention between 7 am and 4 pm on any day of the week to ensure daylight conditions, to complete the intervention alone, and to spend their time simply taking in their natural environment surroundings and not engaging in any physical activity. This included not reading or looking at electronic devices. Participants who wished to remain in the study then proceeded to a separate survey to enter their email address. After the third week, participants were sent an email with a link to the second questionnaire where they again completed all measures.

Waitlist control group participants were told that they would receive instructions in three weeks' time. After the third week, all participants were sent an email with a link to the second questionnaire to once again complete all the measures.

## 7. Results

### 7.1. Study Completion by Participants

**Figure 1:**
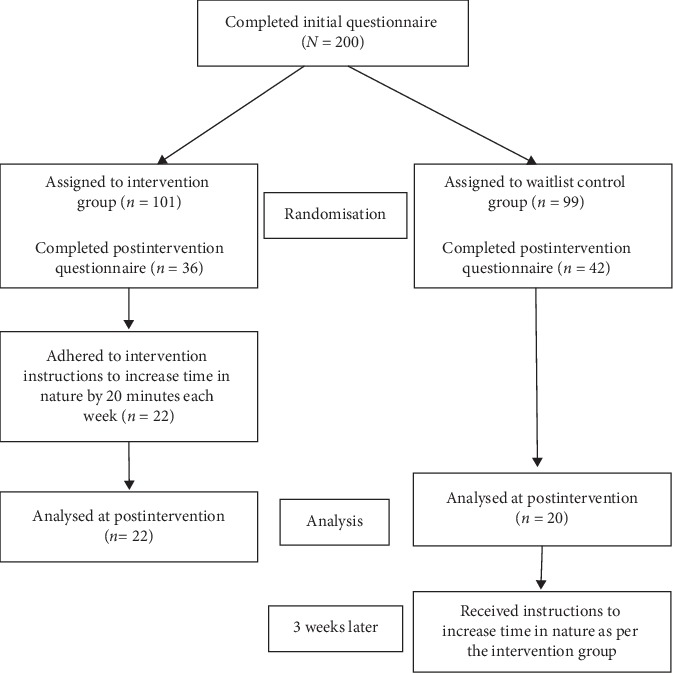
Flow diagram showing study completion and adherence by participants.

Study completion and adherence to the intervention requirements by participants is presented in Figure 1.

### 7.2. Statistical Analysis

All data analysis was carried out in SPSS (version 23.0). Assumption tests were run for all statistical analyses. Inspection of the skewness, kurtosis, and Shapiro–Wilk statistics indicated that the assumptions of normality, linearity, and homoscedasticity were not violated.

### 7.3. Bivariate Correlation Analyses

To assess the size and direction of the linear relationship between restorativeness and well-being, and restorativeness and distress, a bivariate Pearson's product-moment correlation coefficient (*r*) was calculated. Restorativeness was significantly and positively related to well-being, displaying a medium effect size. Restorativeness was also significantly and negatively related to stress and burnout, displaying medium effect sizes according to [40]. Hypothesis 1 was thus supported, and the results are presented in Table 2.

### 7.4. One-Way Covariance Analyses

The means and standard deviations of the PSS, MBI-SS, and SWLS scores for participants who completed the postintervention questionnaire are presented in Table 3. A one-way ANCOVA was used to compare student distress and well-being in the experimental group versus the waitlist control group. Participants' prescores were included as a covariate to partial out the effect of stress, burnout, and life satisfaction levels at the beginning of the intervention period. The ANCOVA indicated that, after accounting for preintervention scores, there was a significant effect of the intervention on stress (*F*(1, 39) = 6.66, *p*=0.014, partial *η*^2^ = 0.17). However, the intervention had no effect on burnout (*F*(1, 39) = 1.23, *p*=0.274, partial *η*^2^ = 0.03) or life satisfaction scores (*F*(1, 39) = 0.10, *p*=0.751, partial *η*^2^ = 0.003). Thus, the hypothesis predicting that those in a natural environment exposure group would experience decreased distress and increased well-being, compared to those in a waitlist control group (after controlling for prescores) was only partially supported. However, there were significant differences between prescores and postscores on measures of stress (*F*(1, 39) = 33.25, *p* < 0.001, partial *η*^2^ = 0.46), burnout (*F*(1, 39) = 145.09, *p* < 0.001, partial *η*^2^ = 0.79), and life satisfaction (*F*(1, 39) = 101.94, *p* < 0.001, partial *η*^2^ = 0.72), with participants in the natural environment group reporting reduced stress and burnout and increased life satisfaction relative to those in the control group.

## 8. Discussion

This study investigated whether spending time in nature would increase reported levels of psychological health in university students. We first hypothesized that higher levels of restorativeness would correlate positively with well-being (life satisfaction) and negatively with distress (stress and burnout). Secondly, it was hypothesized that experimental participants who spent time relaxing in the natural environment would show decreased levels of distress and increased levels of well-being, compared to those in a waitlist control group (after controlling for prescores).

The first hypothesis was supported with a significant positive correlation between restorativeness and well-being and a significant negative correlation between restorativeness and distress. This indicates that increased feelings of restorativeness were associated with increased feelings of satisfaction with one's life. The findings also suggest that individuals who experienced increased levels of restorativeness also experienced decreased levels of stress and burnout. Previous studies have reported a positive relationship between connectedness with nature and psychological well-being [17]. However, according to a literature review examining the role of nature in coping with psychophysiological stress, the relationship between perceived restorativeness and stress measures has not yet been firmly established [11]. There is still evidence to suggest that a significant negative relationship exists, though (e.g., [3]). Future studies may consider exploring a causal relationship between restorativeness and psychological health variables. This would examine whether increased levels of restorativeness predict increased life satisfaction and decreased distress in a university student population.

With respect to the second hypothesis, participants in the experimental group who spent time relaxing in the natural environment showed a significant decrease in stress levels but not in burnout, nor did they experience a significant increase in life satisfaction, compared to those in a waitlist control group (after controlling for prescores). The consensus in the existing literature is that exposure to nature results in a significant increase in psychological health indices (e.g., [2, 3, 9, 16]). For instance, van den Berg et al. [32] found a significant improvement in negative mood, which included stress, after viewing photo and video presentations of a natural environment. Past studies have also found that participants experienced increased subjective well-being and were significantly happier when in green or natural habitats compared to urban environments [41].

In the current study, there was a significant difference in both groups between preintervention and postintervention scores for stress, burnout, and life satisfaction. The waitlist control group experienced a greater mean increase in satisfaction with life compared to the experimental participants. However, when looking at the theoretical frameworks, SRT and ART, there is a core focus on mental fatigue and physiological stress. Experimental participants experienced a larger mean decrease in stress and burnout levels compared to waitlist control participants. This supports the notion that natural environments are generally more restorative than urban or artificial environments [42]. However, in this instance, we compared spending time in a natural environment to *not* spending time in a natural environment. It may thus be of interest for future studies to compare psychological health outcomes after exposure to a natural environment versus urban environment versus waitlist control condition.

### 8.1. Limitations and Future Directions

There are several limitations that should be considered when interpreting the results of the present study.

Firstly, experimental participants chose their own green environment for the intervention. This makes it difficult to determine whether all green environments were equally restorative, or whether some may have been more or less restorative than others. However, other experimental studies have found few differences in restorative impact between different types of natural settings (e.g., [3]), excluding studies that compared extreme (very dense and wild) natural settings [32]. Participants may have also needed to spend a longer period of time in their chosen green environment for a significant change in burnout and life satisfaction to occur. For instance, a study by Tyrväinen et al. [43] indicated that individuals' positive feelings were stronger, compared to those who used green areas less or not at all, when green areas were used for more than five hours per month. However, other studies have found that significant positive effects can occur after spending as little as 15 minutes in nature (e.g., [39]). Future studies may consider extending the time spent in the natural environment.

We also acknowledge that requiring participants to read a description about a character who experienced reduced feelings of stress and burnout and increased feelings of life satisfaction after spending time in a natural environment may have influenced some individuals to expect that engaging in the study would have a positive effect prior to actually undertaking the intervention (i.e., increased the risk of a placebo effect). Future studies need to either control for this issue or incorporate it into their design.

Given the intervention was completed online, the researchers were unable to monitor adherence or ensure that participants fully understood the intervention instructions as would have been the case with a paper-and-pencil intervention. The study relied on participants' self-reports of how many minutes they had spent in their chosen natural environment. Participants were, however, asked to set a physical or electronic reminder, and this may have resulted in a relatively accurate record of their time spent in nature. Future studies could possibly employ apps on smartphones to help assess adherence.

Furthermore, we did not ask waitlist control participants what they did in the intervening three weeks before they received their instructions. As such, we cannot state whether or not these participants spent any time in a natural environment which could then have been controlled for in our analyses.

Finally, although 200 respondents completed the initial questionnaire, only 42 participants were analysed at postintervention. Given the lack of power, the chance of detecting a true effect was reduced. Additionally, while there were 163 females in the final sample, only 37 males participated (with only six males being represented at postintervention). This is relevant as there may be differences in the way that females and males experience distress and well-being. For instance, Ward Thompson et al. [44] found that perceptions of woodlands differ according to age and sex. Specifically, females felt more positively about factors such as areas of open space and were more concerned with visiting woodlands alone and the social stigma that may come with this. However, we acknowledge that this skew in gender representativeness is problematic with respect to the generalisability of the results. Finally, with respect to age, there is a propensity in Australia towards older, often off-campus cohorts who may have a completely different experience while studying at university (e.g., different life stressors, different motivations for study, different support networks, and greater feelings of isolation; [45, 46]). The mean age of the current sample was 31.20 years while similar studies have reported a considerably lower mean age (e.g., 22.2 years in [32] and 21.3 years in [39]).

Increasing psychological health in university students through exposure to nature may provide insight for new interventions, aimed at mitigating student stress and burnout and increasing life satisfaction, to be developed. This would allow for a cost- and time-effective means of enhancing psychological health and would have significant positive implications for university students and higher educational institutions. However, further research is still needed to provide support for the implementation of such interventions.

### 8.2. Barriers to Spending Time in Nature

It is important to consider a range of barriers that may prevent or impact on an individual's experience in natural areas, as this is not generally considered in experimental studies. In their paper, Milligan and Bingley ([47]; p. 809) argued that the notion that “the natural environment is therapeutic” cannot be accepted without some criticism. One study determined that freedom from rubbish and proximity to woodlands were significant factors affecting woodland use [44]. People who live in highly populated urban areas or low socioeconomic areas may not be able to access natural spaces for a number of reasons (e.g., they do not own a motor vehicle or there is no public transport to a nearby natural area). The current study comprised participants who lived in both urban and rural areas, and thus, factors such as accessibility to nature could have been a deterrent for some individuals while completing the intervention. Such barriers should be considered when designing prospective studies.

## 9. Conclusions

The current research was a pilot study within the field of environmental psychology and may provide useful cues for future research. Focusing on student psychological health is particularly valuable as dropping out of university [14], diminished focus, and poor academic performance [18] are all linked to reduced psychological health in university students. The present study suggests that increased restorativeness is associated with increased life satisfaction and decreased stress and burnout. The intervention appears to have helped reduce stress among the experimental participants.

## Figures and Tables

**Table 1:** Study participant characteristics (*N* = 200).

Characteristic	Intervention group (*n* = 101)	Control group (*n* = 99)
Sex		
Male	15 (14.9%)	22 (22.2%)
Female	86 (85.1%)	77 (77.8%)
Age in years, *M* (SD)	31.19 (11.98)	31.20 (11.76)

Year in course of study		
First	32 (31.7%)	26 (26.3%)
Second	11 (10.9%)	15 (15.2)
Third	19 (18.8%)	20 (20.2%)
Honours	23 (22.8%)	16 (16.2%)
Masters	9 (8.9%)	11 (11.1%)
PhD	1 (1.0%)	2 (2.0%)
Others	6 (5.9%)	9 (9.1%)

Location		
Rural	35 (34.7%)	29 (29.3%)
Urban	63 (62.4%)	63 (63.6%)
Others	3 (3.0%)	7 (7.1%)

**Table 2:** Correlations between restorativeness, stress, burnout, and life satisfaction (*N* = 200).

	1	2	3
1. Restorativeness	—		
2. Stress	−0.35^*∗∗*^	—	
3. Burnout	−0.23^*∗∗*^	0.54^*∗∗*^	—
4. Life satisfaction	0.30^*∗∗*^	−0.56^*∗∗*^	−0.37^*∗∗*^

^*∗∗*^ *p* < 0.01 (two-tailed).

**Table 3:** Means and standard deviations of participants who completed the postintervention measures.

	Experimental group (*n* = 22)			Control group (*n* = 20)		
Measure	Preintervention	Postintervention	Hedges' *g* [CI_95%_]	Preintervention	Postintervention	Hedges' *g* [CI_95%_]
Stress (PSS)	21.59 (7.96)	16.59 (6.98)	−0.66 [−1.26, −0.05]	20.35 (6.61)	19.75 (6.08)	−0.09 [−0.71, 0.53]
Burnout (MBI-SS)	2.29 (1.19)	2.03 (1.38)	−0.20 [−0.79, 0.39]	2.30 (0.95)	2.23 (0.99)	−0.07 [−0.69, 0.55]
Life satisfaction (SWLS)	21.64 (7.61)	22.86 (7.80)	0.16 [−0.44, 0.75]	24.10 (5.78)	24.65 (6.03)	0.09 [−0.53, 0.71]

## Data Availability

The data used to support the findings of this study have been deposited in the figshare repository (https://doi.org/10.6084/m9.figshare.6399971.v1).
